# Regulation Mechanism of ssDNA Aptamer in Nanozymes and Application of Nanozyme-Based Aptasensors in Food Safety

**DOI:** 10.3390/foods11040544

**Published:** 2022-02-14

**Authors:** Lijun Wang, Hong Zhou, Haixia Hu, Qin Wang, Xianggui Chen

**Affiliations:** 1 School of Food and Bioengineering, Xihua University, Chengdu 610039, China; wanglijun@mail.xhu.edu.cn (L.W.); zhouhong19980510@163.com (H.Z.); 212021083200011@stu.xhu.edu.cn (H.H.); 2 Department of Nutrition and Food Science, University of Maryland, College Park, MD 20742, USA

**Keywords:** nanozyme, ssDNA aptamer, regulation mechanism, biosensor, food safety

## Abstract

Food safety issues are a worldwide concern. Pathogens, toxins, pesticides, veterinary drugs, heavy metals, and illegal additives are frequently reported to contaminate food and pose a serious threat to human health. Conventional detection methods have difficulties fulfilling the requirements for food development in a modern society. Therefore, novel rapid detection methods are urgently needed for on-site and rapid screening of massive food samples. Due to the extraordinary properties of nanozymes and aptamers, biosensors composed of both of them provide considerable advantages in analytical performances, including sensitivity, specificity, repeatability, and accuracy. They are considered a promising complementary detection method on top of conventional ones for the rapid and accurate detection of food contaminants. In recent years, we have witnessed a flourishing of analytical strategies based on aptamers and nanozymes for the detection of food contaminants, especially novel detection models based on the regulation by single-stranded DNA (ssDNA) of nanozyme activity. However, the applications of nanozyme-based aptasensors in food safety are seldom reviewed. Thus, this paper aims to provide a comprehensive review on nanozyme-based aptasensors in food safety, which are arranged according to the different interaction modes of ssDNA and nanozymes: aptasensors based on nanozyme activity either inhibited or enhanced by ssDNA, nanozymes as signal tags, and other methods. Before introducing the nanozyme-based aptasensors, the regulation by ssDNA of nanozyme activity via diverse factors is discussed systematically for precisely tailoring nanozyme activity in biosensors. Furthermore, current challenges are emphasized, and future perspectives are discussed.

## 1. Introduction

Food safety has become a major global concern because of its impacts on public health as well as the international food trade. Pathogens, toxins, pesticides, veterinary drugs, heavy metals, and illegal additives are common contaminants in foods [1]. To ensure food safety, various conventional methods have been developed to determine food contaminants, including traditional plate culture methods, chromatographic methods, enzyme-linked immunosorbent assay (ELISA), and inductively coupled plasma mass spectrometry (ICP-MS). Although these methods demonstrate high accuracy and reliability for detecting food contaminants, they are complicated, time-consuming, and expensive, and they require special equipment and specially trained personnel, which are difficult to meet the requirements for to conduct on-site and rapid screening of massive food samples and are difficult to apply in many places, such as developing countries. Thus, it is urgent to develop rapid, sensitive, low-cost screening methods as complementary ways of ensuring food safety.

Recently, the fast-growing area of nanomaterials has brought a revolution in the field of analysis and detection, especially with the discovery of nanozymes [2,3]. Nanozymes usually refer to nanomaterials with enzyme-like activities, including the hydrolase and oxidoreductase families. The hydrolase family of nanozymes consists of nuclease- [4], esterase- [5], phosphatase- [6], and protease-like activity [7], while oxidoreductase activity includes peroxidase-, oxidase-, catalase-, and superoxide-dismutase-like activity [8]. Compared with natural enzymes, such as horseradish peroxidase (HRP), nanozymes have several appealing characteristics, such as low cost, high tolerance to harsh conditions (e.g., extreme pH and temperature), facile surface modification, and multifunctionality [9]. These advantages have attracted increasing research interests over the past decade, in turn elucidating the catalytic mechanism of nanozymes and facilitating the rapid development of the field of nanozymes [10,11,12,13]. So far, various nanomaterials, including noble-metal- and transition-metal-based nanomaterials [14,15,16], carbon-based nanomaterials [17,18], metal–organic framework (MOF)-based nanomaterials [19] and their hybrids, have been found to possess peroxidase-like or/and oxidase-like activity, which can be utilized to directly detect H_2_O_2_, glucose, ascorbic acid, and so on [12,20,21,22]. Achieving the specific detection of food contaminants usually requires the introduction of molecularly imprinted polymers or biorecognition elements, such as antibodies, antimicrobial peptides, bacteriophages, nucleic acid probes, and aptamers [12,23,24,25]. Among these, aptamers as alternatives to conventional antibodies are one of the most popular biorecognition elements due to their low cost, facile chemical synthesis, flexible chemical modification, and high tolerance to pH and temperature [26,27]. Furthermore, specific aptamers for almost all possible targets can be obtained via systematic evolution of ligands by exponential enrichment (SELEX). Up to now, various aptamers were obtained against a wide range of targets, from small molecules such as heavy metals to whole cells such as foodborne pathogens [28,29]. More importantly, aptamers’ complementary strands and amplification products are nucleotide sequences, which can reversibly regulate nanozyme activity [30]. These advantages endow nanozyme-based aptasensors with more diversity and promise for food safety applications, especially novel aptasensors based on the regulation by single-stranded DNA (ssDNA) of nanozyme activity.

In view of the extraordinary properties of aptamers and nanozymes, numerous nanozyme-based aptasensors have been developed, and many papers in this field were published in recent years. Although there were several recently published reviews focused on the application of nanozymes in food safety [11,31,32], to the best of our knowledge, systematic reviews specially focusing on nanozyme-aptamer-based biosensors are still rare. To fill this gap, herein, current advances and future prospects of nanozyme-based aptasensors in food safety are systematically discussed. Some factors that govern ssDNA to regulate nanozyme activity are first discussed. Then, the nanozyme-based aptasensors are classified by the different interaction modes of ssDNA and nanozymes, and their applications in food safety are then highlighted. Finally, challenges and prospects for future research in this field are discussed.

## 2. Factors Affecting the Regulation by ssDNA of Nanozyme Activity

**Figure 1:**
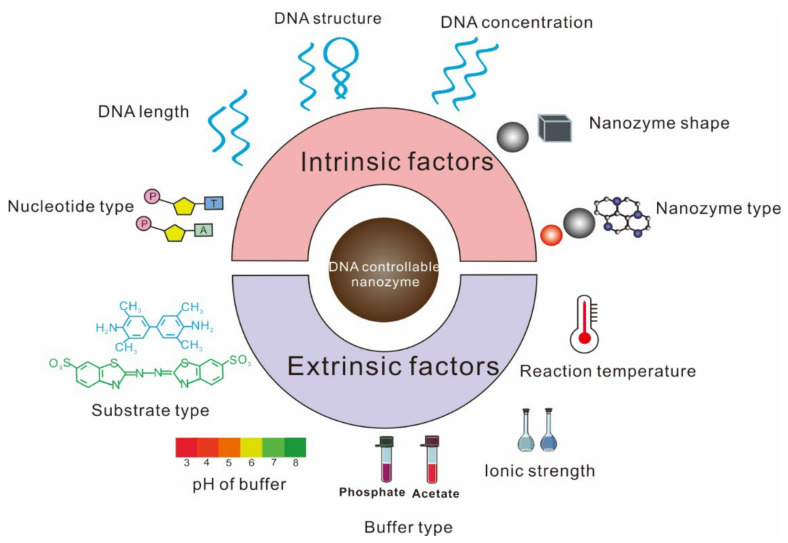
Diverse factors that affect the regulation of ssDNA on nanozyme activity.

The influence of ssDNA on nanozyme activity is complicated. Initially, many research works reported that nanozyme activity was inhibited by ssDNA. As research continued, some groups demonstrated that ssDNA actually improved nanozyme activity [33]. The reasons are yet unclear, but the type of nanozyme and DNA, buffer condition, and temperature in the reaction system were found to be different in these contradicting studies. Hence, to achieve an accurate design of ssDNA-controllable nanozyme biosensors, it is requisite to clearly understand the influence of diverse factors, including intrinsic and extrinsic ones, on the regulation of nanozyme activity by ssDNA (Figure 1).

### 2.1. Factors Affecting ssDNA Inhibition of Nanozyme Activity

Since nanozyme activity is mainly dependent on their surface properties in the solution, bioconjugation or adsorption of ssDNA on the surface of nanozymes might change the surface properties and affect their activity. In general, adsorbed ssDNA could block the surface active sites or induce the aggregation of nanozymes, thus decreasing the binding sites on nanozymes, resulting in decreased activity [34,35]. Thus, the amount of ssDNA aptamer is very important to nanozyme activity. With an increase in aptamer concentration from 2 to 10 μM, the peroxidase-like activity of graphitic carbon nitride@Cu_2_O (g-C_3_N_4_@Cu_2_O) nanocomposites gradually reduced and finally reached a plateau [36], which indicated the saturation of aptamer on the surface of g-C_3_N_4_@Cu_2_O when the amount of aptamer increased to 10 μM. Similar variation trends were observed using Hemin@MOFs nanozyme [37], gold nanoparticles (AuNPs) nanozyme [38], and MnCo_2_O_4_ nanozyme [39]. Different nucleotide types demonstrated different inhibition ability towards nanozyme activity. The order of inhibiting effects towards MnCo_2_O_4_ nanozyme activity was: A > G > C > T [39]. For Mn_3_O_4_ nanozyme, purine nucleotide (A, G) had more active groups to react with octahedral Mn_3_O_4_ nanozymes for blocking surface active sites than pyrimidine nucleotide (C, T), demonstrating a stronger inhibitory effect on the oxidase-like activity of Mn_3_O_4_ [40]. Theoretically, longer ssDNA would have more binding sites to nanozymes and exhibit a better inhibition effect. In fact, 500 nM of poly A_15_ was enough to inhibit the oxidase-like activity of 10 μg/mL MnCo_2_O_4_. Increasing the number of nucleotides from 15 to 45 did not cause an obvious change in MnCo_2_O_4_ nanozyme activity, indicating that a saturation of ssDNA was reached on the MnCo_2_O_4_’s surface [39].

Wang et al. [41] found that double-stranded DNA (dsDNA) did not have the ability to inhibit the oxidase-like activity of a mixed-valence state cerium-based metal–organic framework (MVC-MOF). The difference in ability of ssDNA and dsDNA could be ascribed to the selective adsorption of ssDNA by MVC-MOF [42]. ssDNA has a flexible structure, which contributes to strong π–π stacking interaction between the bases of ssDNA and MOF. Contrarily, dsDNA has a stiff and rigid double-helix structure and cannot wrap around nanomaterials effectively [37], causing low affinity between dsDNA and nanomaterials. However, when the amount of dsDNA was far more than that of ssDNA, the inhibition effect of the former was comparable to or exceeded that of the latter [43,44].

The shape of the nanozyme also affects the interaction of ssDNA and nanozymes. For example, compared with other shapes of Mn_3_O_4_ nanoparticles (i.e., flower-like, polyhedron, spinel), octahedral Mn_3_O_4_ nanozymes had more regular surfaces to interact with ssDNA, resulting in a significant inhibition of oxidase-like activity by ssDNA [40]. Adsorption is the first and key step for realizing DNA regulation of nanozyme activity [45,46]. Different nanomaterials have different kinds of adsorption force to ssDNA [47]. For example, nanoceria adsorbed onto the ssDNA through electrostatic interaction and Lewis acid–base interaction between the phosphate backbone of ssDNA and cerium [35]. The interaction between DNA and AuNPs was tuned by the combination of various noncovalent forces, including electrostatic interactions, hydrophobic forces, and specific bonds between the chemical groups of DNA bases and gold [48]. In addition, the main adsorption force between graphene and ssDNA was π–π stacking interaction [49]. Besides nanozyme type, extrinsic conditions affecting the adsorption forces play an important role in nanozyme activity, including pH and ionic strength. For example, the optimum catalytic condition for MnCo_2_O_4_ nanozyme was pH 3.0, while the nanozyme activity can be maximally inhibited by ssDNA at pH 4.0 [39]. Increasing the ionic strength of buffer weakened the inhibition ability of ssDNA toward MnCo_2_O_4_ nanozyme and enhanced the dissociation ability of ssDNA from the nanozyme’s surface [39].

### 2.2. Factors Affecting ssDNA to Improve Nanozyme Activity

With helps from nanotechnology advancement, researchers have found that ssDNA could enhance the catalytic activity of nanomaterials, including noble-metal-based nanomaterials [33], transition-metal-based nanomaterials, carbon-based nanomaterials [50], MOFs, and their hybrids. As mentioned above, the adsorption of ssDNA onto the surface of nanomaterials was a prerequisite for ssDNA to regulate the nanozyme activity. For example, ssDNA on the surface of nanozymes could adsorb more substrate (e.g., 3,3′,5,5′-tetramethylbenzidine (TMB)) around the nanozyme due to electrostatic attraction and π–π stacking interaction between TMB and ssDNA, further accelerating electron transfer from TMB to the target sensing molecules (e.g., H_2_O_2_) and enhancing the nanozyme activity [45,51]. Thus, factors affecting both ssDNA–nanomaterial and ssDNA–TMB affinity are important for the enhancement of nanozyme activity by ssDNA. The usual trend of nanozyme activity was to first increase and then decrease with the increase in ssDNA concentration [52,53,54]. However, some data support a different variation trend of nanozyme activity, which is to first increase and then keep stable with the increase of ssDNA concentration [46,50,51,55,56]. The reason for the two different trends was unclear. We speculate that the presence of excessive ssDNA may hinder the contact of the nanozyme and TMB in the former trend since it had a wide range of ssDNA concentration. DNA molecules with different structures were also reported to affect the peroxidase-like activity of nanozymes. The order of enhancement of the ability of catalytic activity was: hybridization chain reaction (HCR) products (long dsDNA) > hairpin DNA > ssDNA > dsDNA [53]. Moreover, different DNA nucleotides had different abilities to enhance the nanozyme activity. The order of enhancement of Fe_3_O_4_ nanozyme activity was: C > G > T > A [45]. Cytosine in buffer at pH 4.0 was protonated, which could assist charge neutralization on the surface of Fe_3_O_4_ nanoparticles and reduce repulsion among DNA, leading to the adsorption of more DNA. The same variation trend was observed by Wang et al. [50] using protonated graphitic carbon nitride (Pg-C_3_N_4_) nanosheets as nanozymes. The peroxidase-like activity of 20 bp homo ssDNA (A_20_, C_20_, G_20_, and T_20_) modified MoS_2_ nanozymes followed the trend of G_20_ ≈ T_20_ > A_20_ > C_20_ > no DNA. The explanation from another perspective was given for the weakest activity of C_20_-modified MoS_2_ nanozymes. Protonated cytosine nucleotides in buffer at pH 4.0 increased the electrostatic repulsion between cytosine and positively charged TMB, resulting in a lower affinity of MoS_2_ nanozymes [51]. Guanine (G) was also reported to significantly enhance the peroxidase-like activity of WS_2_ nanosheets [57], iron-based MOFs modified with acidized carbon nanotubes (MOF/CNTs) [52], and MIL-53(Fe) [46]. Purine (A, G) modification demonstrated a remarkable enhancement of the peroxidase-like activity of AuNPs, while pyrimidine (T, C) modification enhanced it slightly, which was attributed to the difference in the interaction between TMB and the surface-adsorbed nucleobases [58]. In addition, the effect of DNA length on the enhancement of nanozyme activity was also investigated. The longest DNA (ploy C_30_) demonstrated the largest enhancement of peroxidase-like activity of Fe_3_O_4_ nanoparticles due to the presence of more binding sites [45]. Increasing the number of cytosine nucleotides from 30 to 40, the peroxidase-like activity of Pg-C_3_N_4_ nanosheets kept steady, which indicated the saturation of ssDNA on the surface of the Pg-C_3_N_4_ nanosheets [50]. A slight inhibition of the catalytic activity of MoS_2_ nanozymes was found when the number of thymidine nucleotides increased from 30 to 40 [51].

Besides the intrinsic factors mentioned above, the reaction conditions were also very important for DNA to regulate the nanozyme activity. The buffer pH at 4.0 had been found to be the optimum condition for both the catalytic activity of nanozymes and the inhibition of nanozyme activity by ssDNA [45,46,56,57]. TMB was the most-used substrate in those literatures. It carries positive charges at pH 4.0 that is below its pKa, which is beneficial to the reaction between ssDNA and TMB [45]. However, continuously reducing the pH resulted in the protonation of two amino acids of TMB, which made them insusceptible to oxidation [59]. Moreover, buffer type and concentration were also reported to affect the ability of ssDNA to enhance the peroxidase-like activity of nanozymes. In a phosphate buffer, low-concentration DNA moderately increased the peroxidase-like activity of CeO_2_, while DNA in acetate buffer had no effect on the catalytic activity of CeO_2_, except for the high-concentration DNA (more than 10 µM), which inhibited the CeO_2_ nanozyme activity [60]. This was because phosphate could compete with ssDNA to bind to CeO_2_ nanozymes and enhance their peroxidase-like activity. Low ionic strength increased the peroxidase-like activity of ssDNA-modified WS_2_ nanosheets [57], while high ionic strength could shield the electrostatic reaction, which reduced the interaction among ssDNA, nanozymes, and TMB [61].

More amazingly, the enhancement or inhibition of the peroxidase-like activity of AuNPs by ssDNA can be controlled by H_2_O_2_ concentration. In a 10 mM H_2_O_2_ reaction system, DNA inhibited the peroxidase-like activity of AuNPs in the first 10 min and enhanced the catalytic activity of AuNPs in the next 20 min of the reaction time, while at a lower concentration of H_2_O_2_ (5 mM), it prolonged the inhibition time to 30 min and enhancement occurred after 30 min [58]. Moreover, by changing the substrate from the positively charged TMB to the negatively charged 2,2′-azino-bis (3-ethyl benzothiazoline-6-sulfonic acid) (ABTS), the enhanced ability of ssDNA disappeared, and the inhibiting ability of ssDNA appeared [45,57].

As previously mentioned, ssDNA can either enhance or inhibit the catalytic activity of nanozymes, and the deciding factor for enhancement or inhibition was to increase or decrease the affinity of substrate (e.g., TMB or ABTS) and nanozymes, respectively, by the introduction of ssDNA. A small change of these factors caused the interaction of ssDNA and nanozymes to be altered, which resulted in variations of the nanozyme activity. Thus, the process of designing aptamer-assisted nanozyme sensing needs to be thoughtful since various factors can affect the regulation of ssDNA on nanozyme activities.

## 3. Nanozyme-Based Aptasensors

In recent years, the application of nanozyme-based aptasensors in food has safety become more and more popular, especially DNA-controllable nanozyme aptasensors. Some of them have been validated in food products, and some have the potential to be used for food samples. In this section, nanozyme-based aptasensors divided into four categories—nanozyme activity inhibited by ssDNA, nanozyme activity enhanced by ssDNA, nanozymes as signal tags, and other methods—are discussed.

### 3.1. Nanozyme Activity Inhibited by ssDNA

In this section, nanozyme activity inhibited by ssDNA was thoroughly discussed, which was dependent on the amount of contaminants in food. Based on the inhibition of nanozyme activity by ssDNA, Sharma et al. [34] developed a “turn-off-on” biosensor strategy based on the peroxidase-like activity of AuNPs and ssDNA Ky2 aptamer for detecting kanamycin. The ssDNA Ky2 aptamer adsorbed onto the surface of AuNPs and blocked the catalytic sites of the AuNPs, resulting in the inhibition of the peroxidase-like activity of AuNPs in the presence of TMB–H_2_O_2_. Conversely, the presence of kanamycin induced the release of ssDNA Ky2 aptamer from the AuNPs due to the specific binding between kanamycin and aptamer. Subsequently, the catalytic activity of the AuNPs recovered, and the solution exhibited a blue color. By measuring the change in absorbance at 650 nm, this aptasensor demonstrated highly sensitive quantitative detection of kanamycin with a limit of detection (LOD) and limit of quantitation (LOQ) of 1.49 nM and 4.52 nM, respectively. This nanozyme-based aptasensor strategy is available for monitoring other food contaminants, such as *Salmonella* typhimurium [36,62], *Campylobacter jejuni* [63], zearalenone [64], sulfadimethoxine [65], chlorpyrifos [66], chloramphenicol [67], acetamiprid [68], Hg(II) [69], norovirus [38], and oxytetracycline [70]. Besides the TMB substrate, the same inhibition phenomenon was observed using negatively charged ABTS as a substrate and AuNPs as nanozymes for detecting streptomycin [71] and acetamiprid [72]. In addition, two convenient chemiluminescence aptasensors were developed for detecting bisphenol A [73] and acetamiprid [74], respectively. In recent year, an ultrasensitive and practical chemiluminescence aptasensor was constructed for detecting acetamiprid by combining the synergistic co-catalysis of graphene oxide (GO)/AuNPs with dexterous interface engineering based on DNA dimensional structure [75]. Due to the high catalytic activity of GO/AuNPs and the perfect regulation of the GO/AuNPs composite interface by DNA dimension, this chemiluminescence sensing demonstrated an ultrahigh sensitivity, with a LOD of 8.9 pM.

However, some researchers found that peroxidase was not the best candidate to study the inhibition effect of ssDNA aptamer on nanozyme activity. This was because the substrate (H_2_O_2_) can generate hydroxyl radicals during the catalytic process which induce DNA cleavage. Considering the high toxicity of the hydroxyl radical to DNA, Wang et al. [41] developed a colorimetric sensor for Hg(II) detection based on the inhibition of the oxidase-like activity of MVC-MOF by thymine-rich ssDNA but not thymine-rich dsDNA. Under optimal conditions, the LOD was 10.5 nM based on S/N = 3, and high selectivity was verified by comparing the signals from other ions’ detection. In another research, octahedral Mn_3_O_4_ nanozymes were also reported to selectively adsorb T-rich ssDNA and were designed to detect Hg(II) and Cd(II) [40]. Using the same nanozyme, Zhu et al. [76] proposed an ultrasensitive colorimetric aptasensor for detecting *S*. *aureus* with a LOD of 3 CFU/mL. Moreover, Huang et al. [39] utilized ochratoxin A (OTA) aptamer to inhibit the oxidase-like activity of MnCo_2_O_4_ for realizing OTA in maize detection, with a LOD of 0.08 mM.

Since TMB is an electrochemically active species, colorimetric methods based on the oxidation of TMB are able to be translated into electrochemical signals for realizing ultrasensitive detection. Das et al. [77] adopted a screen-printed electrode to measure an electrochemically active product via catalyzing TMB oxidation for realizing 60 CFU/mL for detecting *Pseudomonas aeruginosa*. A similar strategy was also proposed for detecting *Escherichia coli* in apple juice in 2 min, with a LOD of 10 CFU/mL [78]. Thionine is also a redox probe and is widely used in electrochemical detection. Wang et al. [79] utilized AuNPs to catalyze the reaction between H_2_O_2_ and thionine to produce oxidized thionine for realizing kanamycin detection with a sensitivity limit of 0.06 nM.

**Figure 2:**
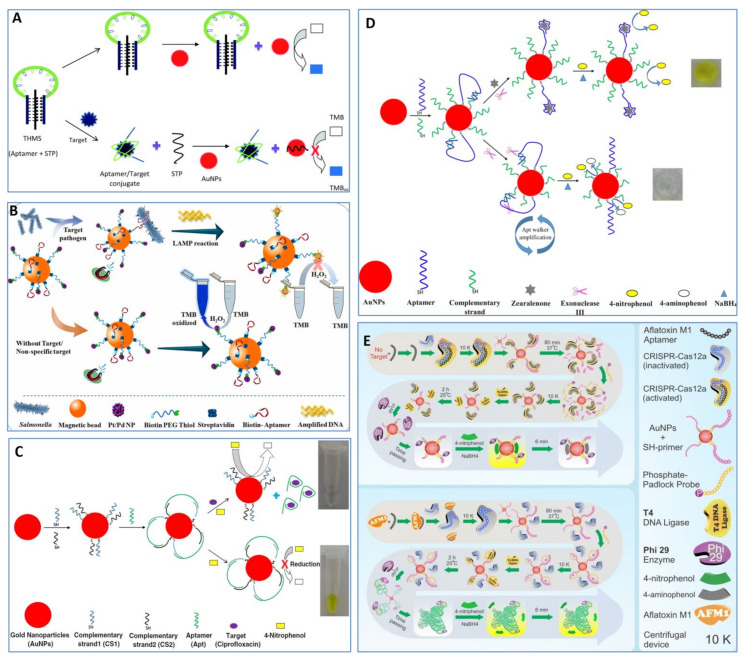
Examples of aptasensor based on the inhibition of nanozyme activity by ssDNA. (**A**) A novel colorimetric triple-helix molecular switch aptasensor for Pb(II) based on the peroxidase-like activity of gold nanoparticles. Reprinted with permission from reference [80]. (**B**) A colorimetric method for *S.* typhimurium based on DNA-mediated inhibition of the peroxidase-like activity of Pt/Pd nanoparticles, loop-mediated isothermal amplification, and magnetic separation. Reprinted with permission from reference [81]. (**C**) A colorimetric aptasensor for ciprofloxacin detection by triggering the reduction-catalyzing activity of gold nanoparticles. Reprinted with permission from reference [82]. (**D**) A colorimetric aptasensor for zearalenone detection based on Exo III-assisted aptamer walker and catalytic reaction of AuNPs. Reprinted with permission from reference [83]. Copyright 2018 American Chemical Society. (**E**) A novel colorimetric aptasensor for ultrasensitive detection of AFM1 based on the combination of CRISPR-Cas12a, rolling circle amplification, and catalytic activity of gold nanoparticle. Reprinted with permission from reference [84].

Taghdisi et al. [80] developed a novel aptasensor for Pb(II) detection based on a triple-helix molecular switch (THMS) and the peroxidase-like activity of AuNPs. As shown in Figure 2A, the two arm segments of Pb(II) aptamer reacted with STP sequence through Watson–Crick and Hoogsteen base pairings to form THMS, which could not adsorb onto the surface of AuNPs. Thus, the AuNPs, with their intrinsic peroxidase-like activity, catalyzed TMB to produce a blue color with the help of H_2_O_2_. However, the presence of Pb(II) caused the formation of Pb(II)–aptamer complexes, and the released STP was able to adsorb onto the surface of the AuNPs to inhibit their peroxidase-like activity. The proposed aptasensor achieved a LOD of 602 pM in a pure aqueous solution. Furthermore, this biosensor was validated in tap water and serum with LODs of 0.708 and 2.07 nM, respectively. Recently, Dehghani et al. [81] integrated magnetic separation, loop mediated isothermal amplification (LAMP), and Pt/Pd nanozymes into one method for on-site detection of *S.* typhimurium (Figure 2B). Dual-functional magnetic beads were first constructed by modifying both *S.* typhimurium aptamers and polyethylene glycol (PEG)-coupled Pt/Pd nanozymes, which were capable to capture bacteria using specific aptamers and output detection signals using the Pt/Pd nanozymes. After magnetic separation, abundant target DNA of *S.* typhimurium was obtained by LAMP to adsorb onto the surface of the Pt/Pd nanozymes, leading to the inhibition of Pt/Pd nanozyme activity. This unique method could detect 10–15 CFU/mL of bacteria in a chicken meat sample and 3–10 CFU/mL of bacteria in both whole egg and chicken fecal samples within less than 3 h.

In addition, AuNPs can also catalyze 4-nitrophenol to produce 4-aminophenol in the presence of NaBH_4_, resulting in the color change of the solution from yellow to colorless. Combining with the catalytic activity of AuNPs, Lavaee et al. [82] developed a colorimetric aptasensor for fluoroquinolones based on the inhibition of catalytic activity of AuNPs by flower-shaped structures. As shown in Figure 2C, both complementary strand 1 of the aptamer (CS1) and complementary strand 2 of the aptamer (CS2) were modified on the surface of AuNPs via Au–S bond. Then, both ends of the aptamer were hybridized with CS1 and CS2, forming a flower-shaped structure on the surface of AuNPs to inhibit the catalytic activity of the AuNPs. However, the presence of ciprofloxacin led to release of the aptamer from the AuNPs, thus resuming the catalytic activity of the AuNPs. By validating in spiked samples, the aptasensor demonstrated high sensitivity, with LODs of 1.3, 2.6, and 3.2 nM in spiked water, serum, and milk samples, respectively, with 1 h analysis time. Subsequently, a colorimetric aptasensor for zearalenone detection was developed by integrating nontarget-induced aptamer walker, the peroxidase-like activity of AuNPs, and exonuclease-assisted recycling amplification [83]. As shown in Figure 2D, both aptamers and the complementary strands were modified on the surface of AuNPs and hybridized with each other to form dsDNA between a portion of aptamer nucleotides and the complementary strand. Then, Exo III nucleases degraded the blunt 3′-terminus of the complementary strand, and the released aptamers bound to other complementary strands, followed by the second degradation process. After numerous degradation processes, the size of the ssDNA-modified AuNPs reduced. Finally, the 4-nitrophenol easily touched the exposed surface of AuNPs and was reduced with the help of NaBH_4_, resulting in the color change of the solution from yellow to colorless. In the presence of zearalenone, the aptamers bound to zearalenone, and the complementary strands could not be degraded. Only a small amount of 4-nitrophenol reached the surface of the AuNPs due to the steric hindrance of aptamer–zearalenone complexes and intact complementary strands on the surface of AuNPs. The solution remained yellow. By measuring the absorbance at 400 nm, the proposed aptasensor detected zearalenone in a wide linear dynamic range from 20 to 80,000 nM, with a LOD of 10 nM.

In addition, Abnous et al. [84] integrated CRISPR-Cas12a, rolling circle amplification, and the catalytic activity of AuNPs into a colorimetric aptasensor for ultrasensitive detection of aflatoxin M1 (AFM1). As shown in Figure 2E, in the presence of AFM1, aptamers cannot bind to the inactivated CRISPR-Cas12a due to aptamer-AFM1’s specific binding. Then, the inactivated CRISPR-Cas12a cannot conduct trans-cleavage of primer. Intact primer-modified AuNPs went through two enzyme reactions (T4 DNA ligase and phi29 DNA polymerase) to yield abundant and long DNA structures on the surface of AuNPs, which hindered the entry of 4-nitrophenol to the surface of the AuNPs. Thus, the solution remained yellow. When AFM1 was absent, CRISPR-Cas12a was activated through the combination of aptamer and CRISPR-Cas12a, which degraded the primer modified on the surface of the AuNPs. Consequently, long ssDNA could not be produced on the surface of AuNPs through two enzyme reactions. Finally, 4-nitrophenol was easily able to reach the surface of the AuNPs, and the solution changed from yellow to colorless. The novel aptasensor achieved a LOD of 0.05 nM in a pure aqueous solution and 0.15 nM in a milk sample. The nanozyme-based aptasensors based on the inhibition of nanozyme activity by ssDNA are listed in Table 1.

As mentioned above, enormous efforts have been made to develop aptasensors based on the inhibition of nanozyme activity by ssDNA for the detection of food contaminants. However, many of the components in food matrixes can adsorb onto the surface of nanozymes and inhibit the peroxidase-like activity of the nanozymes, resulting in low sensitivity or even false positive results for these aptasensors. High-efficiency sample pretreatment incorporated into the nanozyme-based aptasensor, which can minimize the influence of food components on the nanozyme activity, would be a good strategy for high-sensitivity detection of food contaminants.

### 3.2. Nanozyme Activity Enhanced by ssDNA

**Figure 3:**
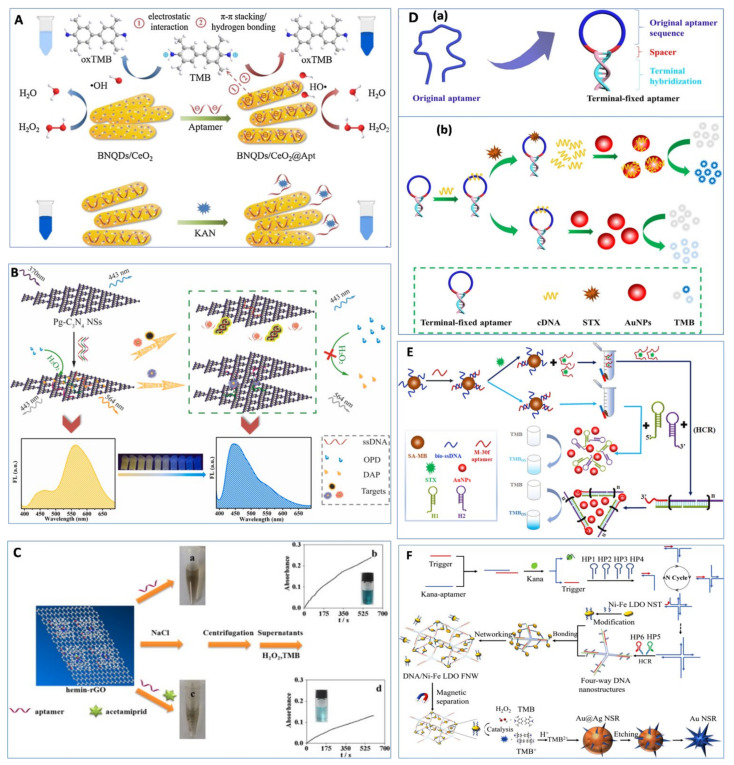
Examples of aptasensor based on the enhancement of nanozyme activity by ssDNA. (**A**) A colorimetric method for kanamycin detection based on the enhancement of the peroxidase-like activity of BNQDs/CeO_2_ by aptamer. Reprinted with permission from reference [56]. (**B**) A versatile ratiometric fluorescence platform based on the enhancement of peroxidase-like activity of Pg-C_3_N_4_ nanosheets by ssDNA. Reprinted with permission from reference [50]. (**C**) A facile label-free colorimetric aptasensor for acetamiprid detection based on the peroxidase-like activity of hemin-rGO. Reprinted with permission from reference [91]. (**D**) A colorimetric detection for saxitoxin based on a terminal-fixed aptamer and the peroxidase-like activity of AuNPs. Reprinted with permission from reference [95]. (a) The design of the terminal-fixed aptamer, (b) Principle of the colorimetric biosensor. (**E**) A competitive colorimetric aptasensor transduced by HCR-facilitated catalysis of AuNPs nanozyme for saxitoxin detection. Reprinted with permission from reference [96]. (**F**) Kanamycin detection based on the etching of Au@Ag nanostars by DNA/Ni–Fe LDO FNW. Reprinted with permission from reference [97].

Aside from those mentioned above, various aptasensor strategies based on the enhancement of nanozyme activity by ssDNA have been proposed. Zhu et al. [56] used aptamers to enhance the peroxidase-like activity of boron nitride quantum dots-anchored porous CeO_2_ nanorods (BNQDs/CeO_2_) to achieve 4.6 pM kanamycin detection (Figure 3A). The BNQDs/CeO_2_ nanozymes showed peroxidase-like activity, while integrating BNQDs/CeO_2_ nanozymes with kanamycin aptamers demonstrated obviously enhanced catalytic activity. However, the presence of kanamycin could inhibit such catalytic enhancement due to the specific binding between aptamer and kanamycin. Thus, the detection signal was negatively correlated with the concentration of the target. Similar mechanisms were also applied for the detection of tetracycline [85], kanamycin [57], sulfadimethoxine [86], cocaine [87], abrin [88], malachite green [89], and *Streptococcus pneumoniae* [90]. In addition, Wang et al. [50] developed a versatile ratiometric fluorescence platform for detecting Hg(II) and aflatoxin B1 (AFB1) based on the enhancement of peroxidase-like activity of Pg-C_3_N_4_ nanosheets by ssDNA. As shown in Figure 3B, Pg-C_3_N_4_ nanosheets had a maximum fluorescence emission of 443 nm under excitation at 370 nm. Subsequent adsorption of aptamer onto the surface of Pg-C_3_N_4_ nanosheets did not change the fluorescence intensity but greatly improved their peroxidase-like activity. The high peroxidase-like activity of aptamer/Pg-C_3_N_4_ complexes could accelerate the catalysis reaction between o-phenylenediamine (OPD) and H_2_O_2_ to produce more 2,3-diaminophenazine (DAP), which quenched the fluorescence of aptamer/Pg-C_3_N_4_ complexes at 443 nm and generated a new emission peak at 564 nm. However, the presence of target molecules induced the dissociation of aptamers from Pg-C_3_N_4_ nanosheets, resulting in low peroxidase-like activity of the Pg-C_3_N_4_ nanosheets. Then, the fluorescence intensity increased at 443 nm, and the fluorescence intensity decreased at 564 nm. By measuring the change in the fluorescence intensity ratio (I_443_/I_564_), the versatile ratiometric fluorescence platform achieved 0.01 nM Hg(II) and a 0.01 pg/mL AFB1 detection limit using AFB1 aptamer. Yang et al. [91] developed a facile label-free colorimetric aptasensor for acetamiprid detection based on the peroxidase-like activity of hemin-functionalized reduced graphene oxide (hemin-rGO) (Figure 3C). Aptamers adsorbed onto the surface of hemin-rGO had an ability to increase the individual hemin-rGO electrostatic repulsion and hinder the coagulation of hemin-rGO in the presence of salt. Thereafter, all hemin-rGO in the supernatant catalyzed TMB in the presence of H_2_O_2_ to produce a blue color. However, the presence of acetamiprid led to the formation of acetamiprid–aptamer complexes, preventing the aptamers from adsorbing onto hemin-rGO. Therefore, hemin-rGO aggregated under salt stress conditions, and little hemin-rGO remained in the supernatant after centrifugation. Thus, the color of the supernatant did not change. Under optimized conditions, the LOD reached 40 nM. Similar methods were reported to detect bisphenol A using hemin-rGO nanozymes [92], as well as Hg(II) and Pb (II) using the peroxidase-like activity of GO–AuNP nanohybrids [93]. Moreover, RNA aptamers were also found preventing the hexadecyltrimethyl ammonium bromide (CTAB)-induced aggregation of AuNPs and improving the peroxidase-like activity of AuNPs. Utilizing this characteristic, a colorimetric aptasensor based on the inhibition of the peroxidase-like activity of AuNPs by CTAB was developed for the detection of malachite green, with a LOD of 1.8 nM [94].

Recently, Li et al. [95] developed a colorimetric detection for saxitoxin based on a terminal-fixed aptamer with enhanced peroxidase-like activity of AuNPs by ssDNA (Figure 3D). A terminal-fixed aptamer was engineered by terminal hybridization (Figure 3D(a)), which was verified as having 145-fold enhancement of binding affinity to saxitoxin compared with the original aptamers. Then, terminal-fixed aptamers were hybridized with a complementary DNA (cDNA) to form terminal-fixed aptamer–cDNA complexes, which could not adsorb onto the surface of AuNPs. In the presence of saxitoxin, the cDNA was released from the terminal-fixed aptamer due to the specific binding between terminal-fixed aptamer and saxitoxin, followed by adsorption onto the surface of AuNPs and enhancement of the peroxidase-like activity of AuNPs. Based on this principle, the colorimetric aptasensor was developed with a LOD of 142.3 pM, and recoveries of 98.21–114.1% in seawater and scallop samples were achieved. In order to improve the sensitivity for saxitoxin detection, HCR and AuNP nanozymes were integrated into one aptasensor developed by the same group [96]. As shown in Figure 3E, cDNA-modified magnetic bead (MB) was hybridized with saxitoxin aptamers (M-30f) to form MB–cDNA–aptamer complexes. In the presence of saxitoxin, aptamers preferred to bind with saxitoxin and released from cDNA-MB. Then, the aptamer–saxitoxin complexes in the supernatant were collected through magnetic separation, serving as a trigger for the HCR to obtain long dsDNA products with a 10-base sticky fragment. With the addition of AuNPs, long dsDNA products adsorbed onto the surface of AuNPs via the 10-base sticky fragment, restricted a certain amount of AuNPs in the limited space, and increased the affinity of TMB to the dense AuNP nanozymes, resulting in the enhancement of the peroxidase-like activity of AuNPs. Without saxitoxin, the supernatant did not contain aptamers after magnetic separation, which hindered the HCR process and could not enhance the catalytic activity of AuNPs. Under optimum conditions, the AuNP-nanozyme-based aptasensor achieved a LOD of 42.46 pM with a linear detection range of 78.13–2500 pM.

Recently, an ultrasensitive quad-amplification strategy for the detection of kanamycin was developed based on three-dimensional DNA/nickel–iron (Ni–Fe) layered double oxide (LDO) frame network-induced “cusp-exposure” of Au@Ag nanostars [97]. As shown in Figure 3F, in the presence of kanamycin, the trigger was released from the aptamers due to the specific binding between kanamycin and aptamer, followed by catalytic hairpin assembly (CHA) and HCR to produce four-way DNA nanostructures. Then, the four-way DNA nanostructures were connected with Ni–Fe LDO to form a DNA/Ni–Fe LDO frame network via DNA hybridization. After magnetic separation, the separated DNA/Ni–Fe LDO frame network demonstrated strong peroxidase-like activity to catalyze TMB to produce TMB^2+^, which etched Ag onto the surface of Au@Ag nanostars and resulted in a red shift of the absorbance peak. By measuring the red shift value, this sensor achieved an ultralow LOD of 3 aM and could detect 0.026 fM kanamycin in milk. Table 2 summarizes the nanozyme-based aptasensors based on the enhancement of nanozyme activity by ssDNA.

Up to now, few studies have reported that food components enhanced nanozyme activity. Thus, aptasensors based on the enhancement of nanozyme activity by ssDNA demonstrated more reliable results in comparison with aptasensors based on the inhibition of nanozyme activity by ssDNA. However, the former had a lower signal-to-noise ratio than that of the latter, indicating lower sensitivity of the aptasensors based on the enhancement of nanozyme activity by ssDNA.

### 3.3. Nanozymes as Signal Tags

**Figure 4:**
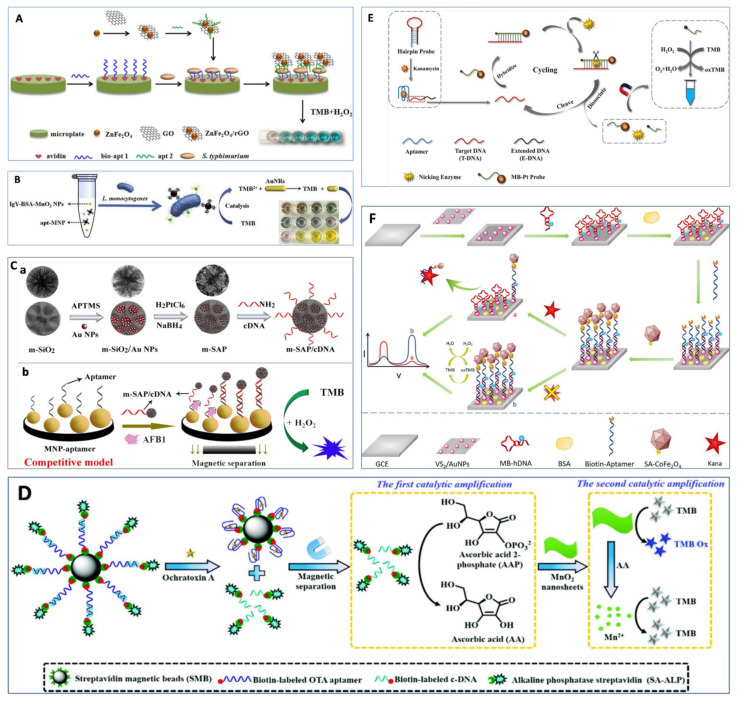
Examples of aptasensor based on nanozymes as signal tags. (**A**) Colorimetric aptasensor for *S.* typhimurium detection using the peroxidase-like activity of ZnFe_2_O_4_-reduced graphene oxide nanostructures [98]. (**B**) A multicolorimetric assay for *L. monocytogenes* detection based on the etching of gold nanorods [102]. (**C**) A nanozyme-based competitive colorimetric aptasensor for AFB1 detection [105]. (a) The synthesis process for m-SAP/cDNA, (b) The principle of nanozyme-based competitive colorimetric aptasensor for AFB1 detection. (**D**) A nanozyme-based cascade colorimetric aptasensor for OTA detection [106]. (**E**) A colorimetric bioassay for detecting kanamycin based on aptamer biorecognition-triggered hairpin switch and nicking-enzyme-assisted signal amplification [107]. (**F**) Ratiometric dual signal-enhancing-based electrochemical biosensor for ultrasensitive kanamycin detection [108]. Copyright 2020 American Chemical Society.

Besides using aptamers to regulate nanozyme activity, another strategy is to apply nanozymes as tags, which aims to establish a proportional relationship between nanozymes and analyte concentrations. The sandwich-type model is one of the most popular approaches to construct highly-sensitive detection of macromolecules, which involves two different recognition molecules binding with target molecules. A colorimetric aptasensor was developed using aptamer immobilized onto a microplate as the capture probe and aptamer-modified ZnFe_2_O_4_/rGO nanozymes as a signal probe for the detection of *S.* typhimurium, and a LOD of 11 CFU/mL was achieved [98] (Figure 4A). Similar research was reported by Duan et al. [99] using aptamer-modified copper-based MOF (Cu-MOF) nanoparticles as signal probes for detecting *E. coli*. In another study, a vancomycin-modified microplate and aptamer-conjugated Fe_3_O_4_ nanoparticle clusters were used to recognize different sites of *L. monocytogenes*, with a LOD of 5.4 × 10^3^ CFU/mL [100]. In order to improve sensitivity and shorten detection time, Liu et al. [101] utilized aptamer-modified MB as a capture probe to extract *L. monocytogenes* from matrix and lgY-antibody-conjugated silver nanoclusters as signal probes to catalyze the substrate OPD, which produced a red color. The method achieved a LOD of 10 CFU/mL without pre-enrichment. Then, the same group also designed another sensitive and reliable multicolorimetric assay for *L. monocytogenes* based on the oxidase-like activity of MnO_2_ and TMB^2+^ etching of gold nanorods (AuNRs) [102]. A sandwich-type model was formed between aptamer-Fe_3_O_4_, *L. monocytogenes*, and lgY-antibody-modified bovine serum albumin (BSA)–MnO_2_ nanozymes. Then, the TMB was catalyzed by lgY–BSA–MnO_2_ to produce TMB^+^ that exhibited blue color. After adding HCl, Au was oxidized to Au(I) and yellow TMB^2+^ was obtained, resulting in a decrease in the aspect ratios of AuNRs, which produced rich-colored optical signals (Figure 4B). Consequently, as little as 10 CFU/mL of *L. monocytogenes* was detected by the naked eye. Wang et al. [103] developed a similar sandwich-type model based on both aptamer-modified MB and Cu-MOF nanozymes for detecting *S. aureus* by measuring residual Cu-MOF nanozymes in the supernatant. The oxidation product of TMB (oxTMB) was inversely proportional to the concentration of *S. aureus.* A LOD of 20 CFU/mL was achieved by this method based on S/N = 3. When using AuNP nanozymes to replace Cu-MOF nanozymes in the sandwich-type model, Yao et al. [104] found that modifying *S. aureus* aptamer on the surface of AuNPs resulted in the inhibition of the peroxidase-like activity of AuNPs. Thus, etching solution (1 µM H_2_O_2_ and 1 µM H_3_PO_4_) was used to enhance the peroxidase-like activity of residual apt-AuNPs in the supernatant after magnetic separation. Finally, the proposed method had a LOD of 10 CFU/mL under optimum conditions.

However, some small molecules have limited binding sites, and it is difficult to find two different recognition molecules to recognize them for constructing biosensors [109]. Thus, an indirect competitive enzyme-linked aptamer assay (ELAA) was developed for detecting tetracycline residues based on the peroxidase-like activity of AuNPs and one aptamer. The study achieved a LOD of 2.7 nM [110]. The tetracycline with a known concentration immobilized on the microplate competed with target tetracycline in honey to bind aptamer-modified AuNPs. Large amounts of tetracycline in honey resulted in small amounts of aptamer-AuNPs binding with the tetracycline immobilized on the microplate, which produced a light blue color in the presence of TMB–H_2_O_2_. Contrarily, a small amount of tetracycline in honey produced a dark blue color.

In addition, aptamer and its cDNA are usually used to design various detection methods for food contaminants. Wu et al. [105] developed a nanozyme-based competitive colorimetric aptasensor for AFB1 detection. As shown in Figure 4C, the aptasensor consisted of aptamer-modified Fe_3_O_4_ magnetic nanoparticles (MNP) as capture probes and cDNA-modified mesoporous SiO_2_/Au–Pt (m-SAP) nanozymes as signal probes. The presence of AFB1 induced the dissociation of cDNA-modified m-SAP nanozyme from MNP, resulting in low absorbance at 650 nm of nanozymes. This aptasensor demonstrated the lowest LOD of 5 nM due to the high peroxidase-like activity of m-SAP. Good selectivity was also confirmed in that the signal for ABF1 was 60 times higher than those of other six mytoxins. In addition, Zhu et al. [111] developed a self-powered photoelectrochemical aptasensor based on the peroxidase-like activity of PtNi nanowires and with benzene-ring-doped g-C_3_N_4_ (BR-CN) as the photoactive material for detecting chloramphenicol. cDNA was first immobilized on a BR-CN-functionalized fluorine-doped tin oxide (FTO) electrode. Then, the biotinylated aptamer (bio-aptamer) was hybridized with cDNA to form biotin-modified dsDNA on the surface of BR-CN/FTO, followed by the addition of streptavidin (SA)-modified PtNi nanozyme to form PtNi-nanozyme-modified dsDNA. PtNi nanozyme catalyzed the oxidation of 4-chloro-1-naphthol (4-CN) by H_2_O_2_ to produce insoluble benzo-4-chlorohexadienone (4-CD) on the electrode surface, resulting in a remarkable decrease in the photocurrent. Contrarily, bio-aptamer was released from the electrode surface in the presence of chloramphenicol. Thus, a lower amount of PtNi nanozyme was captured, resulting in a decrease in catalytic precipitation (4-CD) and an increase in photocurrent signal. By measuring the change in photocurrent intensity, the LOD of 26 fM was obtained with a wide linear range of 0.1 pM–100 nM.

Wang et al. [112] developed a simple colorimetric aptasensor for OTA using Au@Fe_3_O_4_ nanoparticles as a signal indicator and magnetic separator with a LOD of 30 pg/mL. The amino-modified aptamer was immobilized on the surface of glass beads to hybridize with cDNA-modified Au@Fe_3_O_4_. After adding OTA, the aptamers preferred to bind to the OTA, resulting in the dissociation of cDNA-Au@Fe_3_O_4_ from the surface of glass. The released Au@Fe_3_O_4_ nanoparticles were collected by magnetic separation, which catalyzed TMB to produce a blue color in the presence of H_2_O_2_. Similar strategies were also reported to detect AFB1 [113], Cd(II) [114], and chlorpyrifos [115]. On the basis of the methods above, Tian et al. [106] incorporated a cascade reaction (alkaline phosphatase and MnO_2_ nanozymes) into aptasensors for achieving a 0.069 nM LOD of OTA detection (Figure 4D). The ascorbic acid-2-phosphate was catalyzed into ascorbic acid by the released cDNA-modified alkaline phosphatase. Subsequently, the generated ascorbic acid dissolved MnO_2_ nanozymes to generate Mn^2+^ ions, which was not able to catalyze TMB to produce a blue color. Thus, increasing the amount of OTA resulted in the change of color from blue to colorless. On the other hand, Tao et al. [116] utilized the dissociation of cDNA from aptamer-modified MNP by the induction of Pb(II) to inhibit the peroxidase-like activity of graphene/Fe_3_O_4_–Au. The proposed method achieved a LOD of 0.63 µM with the linear range of 1–300 µM. Moreover, Wang et al. [117] developed an ultrasensitive electrochemical aptasensor for detecting Hg^2+^ based on gold–palladium-modified zirconium metal–organic framework (AuPd@UiO-67) nanozyme and T-Hg(II)-T structure, with a LOD of 0.16 nM. Gold-modified thiol graphene (Au@HS-rGO) was immobilized on the electrode, followed by the modification of HS-aptamer 1 on the electrode via Au–S bond. Then, different concentrations of Hg^2+^ were embellished on the aptamer 1. Finally, aptamer-2-functionalized AuPd@UiO-67 was immobilized on the electrode via T-Hg(II)-T structure and DNA hybridization. The current signal could be obtained via the catalytic activity of AuPd@UiO-67 nanozyme toward H_2_O_2_. With the increase in Hg^2+^ concentration, the current signal was enhanced.

Recently, an ultrasensitive colorimetric sensor was developed for detecting kanamycin based on an aptamer biorecognition-triggered hairpin switch and nicking-enzyme-assisted signal amplification, which obtained a LOD of 0.2 pg/mL [107]. As shown in Figure 4E, the hairpin probe contained three parts: kanamycin aptamer, target DNA (T-DNA) located in the hairpin ring, and extended DNA (E-DNA) as an auxiliary stabilizer to the hairpin structure. In the presence of kanamycin, the formed hairpin probe was broken due to the specific binding between aptamer and kanamycin, which prompted the hybridization between the T-DNA and the signal probe. It should be noted that both ends of the signal probe were modified with MB and Pt nanozyme. With the help of nicking enzyme, the signal probe was cleaved into two parts (magnetic bead signal probe and Pt nanozyme signal probe). After magnetic separation, the supernatant containing numerous Pt nanozymes was used to catalyze the oxidation of TMB by H_2_O_2_. Moreover, Tian et al. [108] reported a ratiometric dual signal-enhancing-based electrochemical aptasensor for ultrasensitive detection of kanamycin with a LOD of 0.5 pM (Figure 4F). The VS_2_/AuNPs nanocomposites were modified on the surface of a glassy carbon electrode (GCE), which remarkably accelerated the interface electron transfer. Then, MB-hairpin DNA-SH was coupled with VS_2_/AuNPs/GCE via the Au–S bond, followed by hybridization with the biotin–aptamer to form the double-stranded structure on the electrode. Subsequently, CoFe_2_O_4_ nanozymes conjugated streptavidin bound to biotin–aptamer-catalyzed TMB to produce a strong electronic signal at 0.23 V. In the presence of kanamycin, the aptamer-CoFe_2_O_4_ nanozyme dissociated from the electrode surface, leading to a re-formation of the hairpin DNA. Then, the re-formed hairpin DNA brought MB close to the electrode surface. The electronic signal produced by TMB decreased at 0.23 V, and MB increased greatly at −0.31 V. By measuring the change in the peak current ratio (I_MB_/I_TMB_), this electrochemical aptasensor demonstrated a linear dynamic detection ranging from 1 pM to 1 μM, with a LOD of 0.5 pM. The aptasensors based on nanozymes as signal tags and other methods are presented in Table 3.

Using nanozymes as signal tags in the detection system, especially ELISA, is the typical method used for the quantitative detection of food contaminants and holds great potential to be extended to in-field applications. Simplifying the steps of these aptasensors would accelerate the development of commercialized nanozyme-based aptasensors and should be addressed in future research.

### 3.4. Other Methods Based on Nanozymes and Aptamers

Besides aptamers, *S. aureus* have also been found to inhibit the activity of nanozymes. Zhang et al. [119] developed one-step colorimetric detection of *S. aureus* based on target-induced shielding against the peroxidase-like activity of aptamer-modified Fe_3_O_4_–Au nanocomposites. The color change from dark blue to light blue was proportional to the concentration of *S. aureus.* This colorimetric method was able to detect 10 CFU/mL of *S. aures* by the naked eye in 12 min. The feasibility of this method in five real samples (tap water, Nanhu Lake water, industrial wastewater, urine sample, and milk) was carried out with recoveries between 86.5% and 122.3%. However, a different phenomenon occurred between *S.* typhimurium and gold nanoclusters (AuNCs). Micro-sized *S.* typhimurium captured by aptamer-modified BSA-AuNCs adsorbed more TMB, promoting the affinity between TMB and aptamer-modified BSA-AuNCs. Based on this principle, the developed colorimetric sensor detected *S.* typhimurium with a LOD of 1 CFU/mL [120]. This proposed method was also verified by successful detection of *S.* typhimurium in egg samples, with recoveries between 92.4% and 110%.

## 4. Conclusions and Perspective

Nanozyme-based aptasensors are intensively studied in food safety areas, as is evidenced by a dramatic increase in the number of research articles in the past few years. Due to the extraordinary properties of nanozymes and aptamer, the integration of various nanozymes into aptasensors has significantly improved analytical performance, including sensitivity, selectively, and reproducibility [121]. Compared with conventional ELISA, nanozyme-based aptasensors had some advantages, including high sensitivity and stability and low cost. Thus, nanozyme-based aptasensors are promising alternative tools for rapid detection of food contaminants. Compared with nanozymes serving as signal labels, nanozyme-based aptasensors based on the regulation of nanozyme activity by ssDNA have attracted more attention due to their simplicity, rapidity, and universality. Thus, in this review, the key factors affecting the regulation of nanozyme activity by ssDNA were discussed in detail.

Although a great deal of progress has been made in developing nanozyme-based aptasensors over the past 20 years, many challenges and obstacles still need to be overcome for real-world applications and commercialization. Although various factors were reported to modulate nanozyme activity, the nanozyme activity was still not as good as that of natural nanozymes. Thus, nanozymes with high catalytic activity need to be explored since high catalytic activity would bring high sensitivity of biosensors. Exploiting hybrid nanocomposite enzymes may be a simple strategy for obtaining nanozymes with high catalytic activity. In addition, the mechanism of regulation of nanozyme activity by ssDNA needs to be investigated in depth, because there are still some contradicting points about the relationship between ssDNA and nanozymes. A thorough understanding of the ssDNA regulation mechanism of nanozyme activity would help us to precisely tailor nanozyme activity and further apply it to construct DNA-controllable nanozyme sensing systems. Computer simulation methods may be a good tool to study the regulation mechanism. Finally, as food matrixes are usually complicated, complex sample pretreatment is required to reduce their influence on nanozyme activity to obtain a reliable and sensitive nanozyme-based aptasensor. Thus, high-performance detection with less sample pretreatment would be welcomed in future studies.

## Figures and Tables

**Table 1:** Nanozyme-based aptasensors based on the inhibition of nanozyme activity by ssDNA.

S. No.	Contaminants	Nanozymes	Substrates	Nanozyme Reaction Condition (Buffer, Temperature, Reaction Time)	Sensitivity	Sample Matrix	Linear Range	References
1	Kanamycin	AuNPs	TMB/H_2_O_2_	-	1.49 nM	-	1 to 100 nM	[34]
2	*S*. typhimurium	Fe_3_O_4_ nanoparticles	TMB/H_2_O_2_	0.1 M acetate buffer (pH 4.0), 42 °C, 10 min	7.5 × 10^5^ CFU/mL	-	-	[62]
3	*S*. typhimurium	g-C_3_N_4_@Cu_2_O composites	TMB/H_2_O_2_	-, -, 6 min	15 CFU/mL	Milk	15 to 1.5 × 10^5^ CFU/mL	[36]
4	*C. jejuni*	Au@Pd nanoparticles	TMB/H_2_O_2_	20 mM phosphate buffer (pH 7.0), -, -	100 CFU/mL	Milk	10 to 10^6^ CFU/mL	[63]
5	Zearalenone	AuNPs	TMB/H_2_O_2_	-, 25 °C, 15 min	10 ng/mL	Corn and corn oil	10 to 250 ng/mL	[64]
6	Sulfadimethoxine	AuNPs	TMB/H_2_O_2_	-, -, 10 min	10 ng/mL	Milk	0.01 to 1000 μg/mL	[65]
7	Chlorpyrifos	Tyrosine-capped silver nanoparticles	TMB/H_2_O_2_	37 °C, -, 2 min	11.3 ppm	River water	35 to 210 ppm	[66]
8	Chloramphenicol	Iron-based MOFs	TMB/H_2_O_2_	0.1 M NaAc–HAc buffer (pH 3.0), 40 °C, 20 min	25 nM	Tap water	50 to 200 nM	[67]
9	Acetamiprid	AuNPs	TMB/H_2_O_2_	-, 37 °C, 10 min	0.1 ppm	-	2.5 to 25 ppm	[68]
10	Hg(II)	Fe_3_O_4_ nanoparticles	TMB/H_2_O_2_	0.2 M acetate buffer (pH 4.0), 25 °C, 10 min	5 μM	-	5 to 75 μM	[69]
11	Murine norovirus	AuNPs	TMB/H_2_O_2_	-, -, 10 min	30 viruses/mL	Human serum and shellfish homogenate	200 to 10,000 viruses/mL	[38]
12	Oxytetracycline	Graphene–gold nanoparticle hybrid	TMB/H_2_O_2_	Citrate buffer (pH 4.0), -, -	0.91 nM	-	0.17 to 0.5μM	[70]
13	Streptomycin	AuNPs	ABTS/H_2_O_2_	5 mM sodium acetate (pH 4.5), 30 °C, 10 min	86 nM	Milk	0.1 to 0.5 μM	[71]
14	Acetamiprid	AuNPs	ABTS/H_2_O_2_	3.5 mM NaAc–HAc buffer (pH 5.0), -, -	1.02 μg/L	Wastewater and tomatoes	10 to 160 ng/mL	[72]
15	Bisphenol A	Cationic AuNPs	Luminol/AgNO_3_	-, -, -	62 pg/mL	Soil of an electronic waste dismantling area	0.1 to 40 ng/mL	[73]
16	Acetamiprid	AuNPs	Luminol/H_2_O_2_	-, -, -	62 pM	Wastewater, soil, and cucumber	0.8 to 6.3 × 10^2^ nM	[74]
17	Acetamiprid	GO/AuNPs	Luminol/H_2_O_2_	-, -, -	8.9 pM	Wastewater, soil samples, cucumber, and apple	2.1 × 10^−2^ to 9 nM.	[75]
18	Hg(II)	MVC-MOF nanomaterials	TMB	50 mM NaAc–HAc buffer (pH 4.0), room temperature, 30 min	10.5 nM	Environmental water	0.05 to 6 μM	[41]
19	Hg(II)	Octahedral Mn_3_O_4_ nanoparticles	TMB	25 mM acetate buffer (pH 3.0), -, -	3.8 μg/L	Tap water, river water, lake water, and waste water	10 to 200 μg/L	[40]
	Cd(II)				2.4 μg/L		5 to 100 μM	
20	*S. aureus*	Octahedral Mn_3_O_4_ nanoparticles	TMB	20 mM NaAc (pH 3.5), at room temperature, -	3 CFU/mL	Milk and pork	10 to 2 × 10^5^ CFU/mL	[76]
21	OTA	MnCo_2_O_4_ submicrospheres	TMB	0.1 M acetate buffer (pH 4.0), -, 10 min	0.08 ng/mL	Maize	0.1 to 10 ng/mL	[39]
22	*P. aeruginosa*	AuNPs	TMB/H_2_O_2_	-, -, -	60 CFU/mL	Water	60.0 to 6.0 × 10^7^ CFU/mL	[77]
23	*E. coli*	AuNPs	TMB/H_2_O_2_	-, at room temperature, 2 min	10 CFU/mL	Apple juice	10 to 10^9^ CFU/mL	[78]
24	Kanamycin	AuNPs	Thionine/H_2_O_2_	2 mM Hac–NaAc buffer (pH 4.0), 40 °C, 20 min	0.06 nM	Honey	0.1 to 60 nM	[79]
25	Pb(II)	AuNPs	TMB/H_2_O_2_	-, 37 °C, 5 min	602 pM in pure aqueous solution, 0.708 nM in tap water, 2.07 nM in rat serum.	Water and serum	0.2 to 30 nM	[76]
26	*S*. typhimurium	Pt/Pd nanoparticles	TMB/H_2_O_2_	10 mM Tris buffer saline (pH 8.0), room temperature, 1 min	10–15 CFU/mL in chicken meat, 3–10 CFU/mL in whole egg and chicken feces	Chicken meat, whole egg, and chicken feces	-	[81]
27	Ciprofloxacin	AuNPs	4-nitrophenol/NaBH_4_	-, -, 3 min	1.2 nM in pure aqueous solution, 1.3 nM in milk, 2.6 nM in serum, and 3.2 nM in milk	Spiked water, serum, and milk	4 to 500 nM	[82]
28	Zearalenone	AuNPs	4-nitrophenol/NaBH_4_	-, 23 °C, 7 min	10 ng/L	Human serum sample	20 to 80,000 ng/L	[83]
29	AFM1	AuNPs	4-nitrophenol/NaBH_4_	-, room temperature, 6 min	0.05 ng/L	Milk sample	0.2 to 300 ng/L	[84]

**Table 2:** Nanozyme-based aptasensors based on the enhancement of nanozyme activity by ssDNA.

S. No.	Contaminants	Nanozymes	Substrates	Nanozyme Reaction condition (Buffer, Temperature, Reaction Time)	Sensitivity	Sample Matrix	Linear Range	References
1	Kanamycin	BNQDs/CeO_2_ nanorods	TMB/H_2_O_2_	0.2 M acetate buffer (pH 4.0), 30 °C, 10 min	4.6 pM	Swine urine, milk, and wastewater	0.01 to 100 nM	[56]
2	Tetracycline	AuNCs	TMB/H_2_O_2_	0.2 M NaAc–HAc buffer (pH 3.8), room temperature, 90 min	46 nM (spectrophotometer), 0.5 μM (naked-eye detection)	Milk	1 to 16 μM	[85]
3	Kanamycin	Layered WS_2_ nanosheets	TMB/H_2_O_2_	20 mM NaAc buffer (pH 4.0), -, 50 s	0.06 µM	Milk, honey, and pork	0.1 to 0.5 µM	[57]
4	Sulfadimethoxine	Cu(HBTC)-1/Fe_3_O_4_– AuNPs nanosheets	TMB/H_2_O_2_	0.2 M acetate buffer (pH 4.0), room temperature, 3 min	1.70 μg/L	Tap water	3.57 to 357.14 μg/L	[86]
5	Cocaine	Hemin-functionalized graphene nanosheets	ABTS/H_2_O_2_	50 mM NaH_2_PO_4_ (pH 3.6), -, immediate detection	230 μM	-	0.5 to 5 mM	[87]
6	Abrin	AuNPs	TMB/H_2_O_2_	-, 25 °C, 10 min	0.05 nM	Raw milk	0.2 to 17.5 nM	[88]
7	Malachite green	Fe_3_O_4_ nanoparticles	TMB/H_2_O_2_	20 mM NaAc–HAc (pH 4.0), 37 °C, 10 min	16.7 μg/kg	Fish and water	0.06 to 2.38 μM	[89]
8	*S. pneumoniae*	Citric acid-functionalized silver/copper nanoparticles	TMB/H_2_O_2_	-, 37 °C, 10 min	65 CFU/mL	Milk and fruit juice	10^2^ to 10^8^ CFU/mL	[90]
9	Hg(II)	Pg–C_3_N_4_ nanosheets	OPD/H_2_O_2_	10 mM NaAc–HAc buffer (pH 4.0), 35 °C, 3 min,	0.01 nM	Real Xiangjiang River water from Changsha and tap water from laboratory	0.05 to 100 nM	[50]
	AFB1				0.01 pg/mL	Peanut, maize, and wheat	0.1 to 60 pg/mL	
10	Acetamiprid	Hemin-rGO	TMB/H_2_O_2_	25 mM PBS (pH 5.0), 35 °C, 10 min	40 nM	Wastewater	0.1 to 10 μM	[91]
11	Bisphenol A	Hemin-rGO	TMB/H_2_O_2_	-, -, -	2 nM	Tap water	5 to 100 nM	[92]
12	Hg(II)	Graphene oxide–gold nanohybrids	TMB/H_2_O_2_	33.3 mM sodium acetate buffer (pH 4.3), -, -	300 nM	River water	0 to 50 µM	[93]
	Pb(II)				500 nM			
13	Malachite green	AuNPs	TMB/H_2_O_2_	10 mM NaAc–HAc (pH 4.0), -, 20 min	1.8 nM	Fresh water and seawater	10 to 500 nM	[94]
14	Saxitoxin	AuNPs	TMB/H_2_O_2_	Acetate acid (pH 4.0), -, 15 min.	142.3 pM	Seawater and scallop	0.1457 to 37.30 nM	[95]
15	Saxitoxin	AuNPs	TMB/H_2_O_2_	Acetate acid (pH 4.0), -, 25 °C,	42.46 pM	Scallop	78.13 to 2500 pM	[96]
16	Kanamycin	Ni–Fe LDO	TMB/H_2_O_2_	0.1 M NaAc–HAc (pH 4.0), -, 5 min	3 aM	Milk	0.01 fM to 0.1 nM.	[97]

**Table 3:** Nanozyme-based aptasensors based on nanozyme as signal tag and other methods.

S. No.	Contaminants	Nanozymes	Substrates	Nanozyme Reaction Condition (Buffer, Temperature, Reaction Time)	Sensitivity	Sample Matrix	Linear Range	References
1	*S.* typhimurium	ZnFe_2_O_4_-reduced graphene oxide nanostructures	TMB/H_2_O_2_	NaAc (pH 3.5), -, 20 min	11 CFU/mL	Milk	11 to 1.10 × 10^5^ CFU/mL	[98]
2	*E. coli*	Cu-MOF	TMB/H_2_O_2_	0.2 M acetate (pH 4.0), -, 10 min	2 CFU/mL	Milk	16 to 1.6 × 10^6^ CFU/mL	[99]
3	*L. monocytogenes*	Fe_3_O_4_ nanoparticle cluster	TMB/H_2_O_2_	-, -, -	5.4 × 10^3^ CFU/mL	Milk	5.4 × 10^3^ to 10^8^ CFU/mL	[100]
4	*L. monocytogenes*	Silver nanoclusters	OPD	-, room temperature, 3 min	10 CFU/mL	Pork	10 to 10^6^ CFU/mL	[101]
5	*L. monocytogenes*	MnO_2_	TMB	-, -, -	10 CFU/mL	Pork	10 to 10^6^ CFU/mL	[102]
6	*S. aureus*	Cu-MOF	TMB/H_2_O_2_	-, 45 °C, 10 min	20 CFU/mL	Milk	50 to 10,000 CFU/mL	[103]
7	*S. aureus*	AuNPs	TMB/H_2_O_2_	-, -, 5 min	10 CFU/mL	Pork and milk	10 to 10^6^ CFU/mL	[104]
8	Tetracycline	AuNPs	TMB/H_2_O_2_	Buffer (0.08 M Na_2_HPO_4_·12H_2_O, 0.1 M citric acid), -, 15 min	2.7 pg/mL	Honey	0.01 to 10 ng/mL	[110]
9	AFB1	Mesoporous SiO_2_/Au–Pt	TMB/H_2_O_2_	-, room temperature, 10 min	5 pg/mL	Peanut	0.01 to 1000 ng/mL	[105]
10	Chloramphenicol	PtNi nanowires	4-chloro-1-naphthol/H_2_O_2_	-, room temperature, 20 min	26 fM	Pig urine, river water, and milk	0.1 pM to 100 nM	[111]
11	OTA	Au@Fe_3_O_4_ nanoparticles	TMB/H_2_O_2_	0.2 M acetate buffer solution (pH 4.0), 40 °C, 15 min	30 pg/mL	Cereal	0.5 to 100 ng/mL	[112]
12	AFB1	AuNPs	TMB/H_2_O_2_	0.2 M acetate buffer solution, 40 °C, 20 min	0.43 pg/mL	Corn	5 to 200 ng/mL	[113]
13	Cd(II)	Au–MoS_2_ nanocomposites	TMB/H_2_O_2_	-, room temperature, 5 min	0.7 ng/mL	White wine	1 to 500 ng/mL	[114]
14	Chlorpyrifos	Cu-MOF	TMB/H_2_O_2_	-, 40 °C water bath, 15 min	4.4 ng/mL	Winter jujube, apple, cabbage, and cucumber	0 to 1250 ng/mL	[115]
15	OTA	MnO_2_ nanosheets	TMB	0.2 M NaAc–HAc (pH 4.5), room temperature, 5 min	0.069 nM	Grape juice	1.25 to 250 nM	[106]
16	Pb(II)	Graphene/Fe_3_O_4_–Au nanoparticles	TMB/H_2_O_2_	-, room temperature, 5 min	0.63 ng/mL	Tap water	1 to 300 ng/mL	[116]
17	Hg(II)	AuPd@UiO-67 nanomaterial	H_2_O_2_	-, -, -	0.16 nM	Tap water and lake water	1.0 to 10^3^ nM	[117]
18	Kanamycin	Pt nanoparticles	TMB/H_2_O_2_	-, 45 °C, 10 min	0.2 pg/mL	Milk	0.5 to 2 × 10^5^ pg/mL	[107]
19	Kanamycin	CoFe_2_O_4_ nanoparticles	TMB/H_2_O_2_	0.01 M PBS, -, -	0.5 pM	Milk	1 pM to 1 μM	[108]
20	Parathion	Platinum nanoparticles	TMB/H_2_O_2_	-, -, -	2 pg/mL	Water, pear, cabbage, and rice	0.01 to 50 ng/mL	[118]
21	*S. aureus*	Fe_3_O_4_–Au nanoparticles	TMB/H_2_O_2_	0.2 M acetate acid –sodium acetate buffer (pH 4.0), room temperature, 2 min	10 CFU/mL by eye, 26 CFU/mL by spectrophotometer	Tap water, Nanhu Lake water, industrial wastewater, urine sample, and milk	10 to 10^6^ CFU/mL	[119]
22	*S*. typhimurium	AuNCs	TMB/H_2_O_2_	10 mM PBS (pH 5.5), -, 3 min	1 CFU/mL	Eggshell and egg white	10 to 10^6^ CFU/mL	[120]

## Data Availability

The study did not report any data.

## Abbreviations

ELISA: enzyme-linked immunosorbent assay; ICP-MS, inductively coupled plasma mass spectrometry; HRP, horseradish peroxidase; MOF, metal–organic framework; SELEX, systematic evolution of ligands by exponential enrichment; ssDNA, single-stranded DNA; g-C_3_N_4_@Cu_2_O, graphitic carbon nitride@Cu_2_O; AuNPs, gold nanoparticles; dsDNA, double-stranded DNA; MVC-MOF, mixed-valence state cerium-based metal–organic framework; TMB, 3,3′,5,5′-tetramethylbenzidine; HCR, hybridization chain reaction; pg-C_3_N_4_, protonated graphitic carbon nitride; G, guanine; CNTs, carbon nanotubes; ABTS, 2,2′-azino-bis (3-ethyl benzothiazoline-6-sulfonic acid); LOD, limit of detection; LOQ, limit of quantitation; GO, graphene oxide; OTA, ochratoxin A; THMS, triple-helix molecular switch; LAMP, loop-mediated isothermal amplification; PEG, polyethylene glycol; CS1, complementary strand 1 of aptamer; CS2, complementary strand 2 of aptamer; AFM1, aflatoxin M1; BNQDs, boron nitride quantum dots; AFB1, aflatoxin B1; OPD, o-phenylenediamine; DAP, 2,3-diaminophenazine; hemin-rGO, hemin-functionalized reduced graphene oxide; CTAB, hexadecyltrimethyl ammonium bromide; cDNA, complementary DNA; MB, magnetic bead; Ni–Fe, nickel–iron; LDO, layered double oxide; CHA, catalytic hairpin assembly; Cu-MOF, copper-based MOF; AuNRs, gold nanorods; BSA, bovine serum albumin; ELAA, enzyme-linked aptamer assay; MNP, magnetic nanoparticles; m-SAP, mesoporous SiO_2_/Au–Pt; BR-CN, benzene-ring-doped g-C_3_N_4_; FTO, fluorine-doped tin oxide; SA, streptavidin; 4-CN, 4-chloro-1-naphthol; 4-CD, benzo-4-chlorohexadienone; AuPd@UiO-67, gold–palladium-modified zirconium metal–organic frameworks; Au@HS-rGO, gold-modified thiol graphene; T-DNA, target DNA; E-DNA, extended DNA; GCE, glassy carbon electrode; AuNCs, gold nanoclusters.
